# *Phyllosticta citricarpa* and sister species of global importance to *Citrus*

**DOI:** 10.1111/mpp.12861

**Published:** 2019-09-11

**Authors:** Vladimiro Guarnaccia, Thies Gehrmann, Geraldo J. Silva‐Junior, Paul H. Fourie, Sajeet Haridas, Duong Vu, Joseph Spatafora, Francis M. Martin, Vincent Robert, Igor V. Grigoriev, Johannes Z. Groenewald, Pedro W. Crous

**Affiliations:** ^1^ Westerdijk Fungal Biodiversity Institute Utrecht Netherlands; ^2^ DiSAFA, University of Torino Largo Paolo Braccini 2 10095 Grugliasco TO Italy; ^3^ Fund for Citrus Protection, Fundecitrus Araraquara São Paulo Brazil; ^4^ Citrus Research International P.O. Box 28 Nelspruit 1200 South Africa; ^5^ Department of Plant Pathology Stellenbosch University Private Bag X1 Stellenbosch 7602 South Africa; ^6^ US Department of Energy Joint Genome Institute 2800 Mitchell Dr. Walnut Creek CA 94598 USA; ^7^ Department of Botany and Plant Pathology Oregon State University Cordley Hall 2082 Corvallis 97331‐2902 OR USA; ^8^ Institut National de la Recherche Agronomique, UMR INRA‐Université de Lorraine “Interaction Arbres/Microorganismes” Champenoux France

**Keywords:** *Citrus*, *Guignardia*, mating type, systematics

## Abstract

Several *Phyllosticta* species are known as pathogens of *Citrus* spp., and are responsible for various disease symptoms including leaf and fruit spots. One of the most important species is *P*. *citricarpa,* which causes a foliar and fruit disease called citrus black spot. The *Phyllosticta* species occurring on citrus can most effectively be distinguished from *P*. *citricarpa* by means of multilocus DNA sequence data. Recent studies also demonstrated *P*. *citricarpa* to be heterothallic, and reported successful mating in the laboratory. Since the domestication of citrus, different clones of *P. citricarpa* have escaped Asia to other continents via trade routes, with obvious disease management consequences. This pathogen profile represents a comprehensive literature review of this pathogen and allied taxa associated with citrus, focusing on identification, distribution, genomics, epidemiology and disease management. This review also considers the knowledge emerging from seven genomes of *Phyllosticta* spp., demonstrating unknown aspects of these species, including their mating behaviour.

**Taxonomy:**

*Phyllosticta citricarpa* (McAlpine) Aa, 1973. Kingdom Fungi, Phylum Ascomycota, Class Dothideomycetes, Order Botryosphaeriales, Family Phyllostictaceae, Genus *Phyllosticta*, Species *citricarpa*.

**Host range:**

Confirmed on more than 12 *Citrus* species, *Phyllosticta citricarpa* has only been found on plant species in the Rutaceae.

**Disease symptoms:**

*P. citricarpa* causes diverse symptoms such as hard spot, virulent spot, false melanose and freckle spot on fruit, and necrotic lesions on leaves and twigs.

**Useful websites:**

DOE Joint Genome Institute MycoCosm portals for the *Phyllosticta capitalensis* (https://genome.jgi.doe.gov/Phycap1), *P*. *citriasiana* (https://genome.jgi.doe.gov/Phycit1), *P*. *citribraziliensis* (https://genome.jgi.doe.gov/Phcit1), *P*. *citrichinaensis* (https://genome.jgi.doe.gov/Phcitr1), *P*. *citricarpa* (https://genome.jgi.doe.gov/Phycitr1, https://genome.jgi.doe.gov/Phycpc1), *P*. *paracitricarpa* (https://genome.jgi.doe.gov/Phy27169) genomes.

All available *Phyllosticta* genomes on MycoCosm can be viewed at https://genome.jgi.doe.gov/Phyllosticta.

## Introduction

*Phyllosticta* species have been associated with *Citrus* spp. worldwide (Baayen *et al*., 2002; Baldassari *et al*., 2008; Brentu *et al*., 2012; Carstens *et al*., 2012; Er *et al*., 2014; Everett and Rees‐George 2006; Glienke *et al*., 2011; Glienke‐Blanco *et al*., 2002; Guarnaccia *et al*., 2017a; Wikee *et al*., 2013a; Wulandari *et al*., 2009). Citrus black spot (CBS) is an economically important foliar and fruit disease caused by *P. citricarpa* (Baldassari *et al*., 2008; Kotzé, 1981). The pathogen affects the rind of the fruit, causing different symptoms on almost all commercial citrus cultivars, with some lemons, limes, mandarins and late‐maturing sweet oranges being the most susceptible (Kiely, 1948a, 1948b, 1949; Kotzé, 1981, 2000; Snowdon, 1990). CBS disease was reported for the first time in Australia (Benson, 1895), and is currently present in warm, summer rainfall areas of Asia, Africa, South America and North America (Kotzé, 1981; Schubert *et al*., 2012; Yonow *et al*., 2013).

*Phyllosticta citricarpa* was previously not readily distinguishable from *P*. *capitalensis*, a morphologically similar but nonpathogenic species, considered as the asexual morph of *Guignardia mangiferae* (Baayen *et al*., 2002; Everett and Rees‐George, 2006; Glienke *et al*., 2011). After the advent of DNA‐based characterization, a robust multilocus analysis was developed to distinguish *P*. *citricarpa* from *P*. *capitalensis* and other *Phyllosticta* species, resolving the identification and detection of *P*. *citricarpa* (Glienke *et al*., 2011).

*Phyllosticta capitalensis* is a cosmopolitan fungus reported from a broad range of plant hosts (Baayen *et al*., 2002; Bezerra *et al*., 2012; Everett and Rees‐George, 2006; Glienke‐Blanco *et al*., 2002; Johnston, 1998; Meyer *et al*., 2006; Okane *et al*., 2001; Rakotoniriana *et al*., 2008; Rodrigues and Samuels, 1999; Rodrigues *et al*., 2004; Wikee *et al*., 2011; Yuan *et al*., 2009), and has been found on citrus associated with both CBS‐affected and asymptomatic plants (Baayen *et al*., 2002; Everett and Rees‐George, 2006; Glienke *et al*., 2011). Further *Phyllosticta* species have been reported as pathogens of citrus, namely *P*. *citriasiana* and *P. citrimaxima*, responsible for citrus tan spot disease on *Citrus maxima* in Asia (Wikee *et al*., 2013b; Wulandari *et al*., 2009), *P*. *citrichinaensis* on different citrus species in Asia and *P. paracitricarpa* on *Citrus sinensis* fruit in China (Wang *et al*., 2012). The last species was also detected from citrus leaf litter in Europe (Guarnaccia *et al*., 2017a). Nevertheless, *P*. *citribraziliensis* and *P*. *paracapitalensis* were reported as endophytes on different citrus hosts (Glienke *et al*., 2011; Guarnaccia *et al*., 2017a).

The occurrence of two opposite mating types genes (*MAT1‐1‐1* and *MAT1‐2‐1*) has been reported in *P. citricarpa* populations, confirming the heterothallic behaviour of the fungus and the necessity of crossing between opposite mating types in order to produce viable progeny (Amorim *et al*., 2017; Wang *et al*., 2013, 2016). Carstens *et al*. (2017) conducted the first study on the global mating type distribution of *P. citricarpa*. Despite the importance of homo‐ or heterothallism and knowledge of mating type distribution, little is known about the sexual cycle of most *Phyllosticta* species associated with citrus.

This review focuses on the mating‐type behaviour of *P*. *citricarpa* and other related species associated with citrus, and improved methods for their detection. Currently available knowledge regarding the pathogen’s geographical distribution, disease epidemiology and management is summarized. Furthermore, key insights emerging from the genomes of six *Phyllosticta* spp. recently sequenced are highlighted. The purpose of this review is to provide a basis for future studies aimed at understanding the rapid detection of *Phyllosticta* species associated with citrus, their mating type distribution and global movement.

## Taxonomy and Identification

The name previously applied to the sexual morph of *Phyllosticta* is *Guignardia* (van der Aa, 1973; Rossman *et al*., 2015). *Guignardia* was introduced by Viala and Ravaz (1892) as a replacement for *Laestadia* (Bissett, 1986). *Guignardia bidwellii* and related species were included in *Botryosphaeria* by Petrak (1957), and this proposal was supported by Barr (1970, 1972). Later, van der Aa (1973) monographed the genus *Phyllosticta,* and all species were re‐evaluated by van der Aa and Vanev (2002). Schoch *et al*. (2006) placed *Phyllosticta* in the order Botryosphaeriales. However, several authors showed that Botryosphaeriaceae contained both *Botryosphaeria* and *Phyllosticta* spp., although this relationship remained poorly resolved (Crous *et al*., 2006; Liu *et al*., 2012; Schoch *et al*., 2006).

The older name *Phyllosticta* was retained over that of *Guignardia* after the end of fungal dual nomenclature (Glienke *et al*., 2011; Hawksworth *et al*., 2011; Marin‐Felix *et al*., 2019; Rossman *et al*., 2015; Sultan *et al*., 2011; Wikee *et al*., 2011, 2013b; Wingfield *et al*., 2012; Wong *et al*., 2012; Wijayawardene *et al*., 2014). Wikee *et al*. (2013b) redefined *Phyllosticta* and resurrected the family name Phyllostictaceae, showing that it clusters separately from the Botryosphaeriaceae within the Botryosphaeriales.

The *in vitro* production of pycnidial conidiomata containing aseptate, hyaline conidia, encased in a mucoid layer and bearing a single apical appendage (van der Aa, 1973), is the most characteristic feature used to recognize species of *Phyllosticta*. However, the mucoid layer and appendage are not always present or visible. The sexual morph presents erumpent, globose to pyriform ascomata, often irregularly shaped, unilocular and with a central ostiole. Asci are eight‐spored, bitunicate, clavate to broadly ellipsoid, with a wide, obtusely rounded or slightly square apex. Ascospores are ellipsoid to limoniform, sometimes slightly elongated, aseptate, hyaline, showing a large central guttule and a mucoid cap at both ends. Spermatia produced in culture are hyaline, aseptate, cylindrical to dumbbell‐shaped with guttules at each end (van der Aa, 1973).

Studies incorporating DNA sequence data (Baayen *et al*., 2002; Glienke *et al*., 2011; Wikee *et al*., 2011; Wulandari *et al*., 2009) resolved different *Phyllosticta* species associated with citrus that were described based on phylogenetic data, morphology and culture characteristics (Crous *et al*., 2012; Su and Cai, 2012; Wang *et al*., 2012; Wong *et al*., 2012; Zhang *et al*., 2012).

*Phyllosticta citricarpa* and *P. capitalensis* have several morphological and physiological differences: (i) colonies of *P. citricarpa* produce a yellow halo when grown on oatmeal agar (OA), (ii) *P. capitalensis* generally grows faster, (iii) conidia of *P. capitalensis* are normally embedded within a thicker mucoid sheath than observed in *P. citricarpa*, and (iv) there is a higher level of hydrolytic enzyme production in *P. citricarpa* than in *P. capitalensis* (Baayen *et al*., 2002; Glienke *et al*., 2011; Romão *et al*., 2011). Apart from colony characteristics on OA, morphology is unreliable to distinguish these species, and the identification of *P. citricarpa* has been confused with *P. capitalensis*, which is easily isolated as an endophyte from a broad range of hosts, and commonly co‐occurs with *P*. *citricarpa* on citrus.

*Phyllosticta capitalensis* was originally described on *Stanhopea* (Orchidaceae) from Brazil by Hennings (1908). The name *P. capitalensis* was later applied to an endophytic species occurring in ericaceous plants in Japan by Okane *et al*. (2001), who also described its sexual morph as a new species, *G. endophyllicola*. The name to apply to the sexual morph has been much debated in the past. Baayen *et al*. (2002), based on DNA sequence data of the rDNA internal transcribed spacer (ITS) region, linked *P. capitalensis* to *G. mangiferae*, a species originally reported from *Mangifera indica* in India, separating *P. capitalensis* from *G. endophyllicola*. Later, Glienke *et al*. (2011), on the basis of a multilocus phylogenetic analysis, revealed that *P. capitalensis sensu lato* was genetically distinct from a reference isolate of *G. mangiferae*, and concluded that the correct name for the common endophyte is *P. capitalensis,* since *G. mangiferae* is a distinct taxon on mango. *Phyllosticta citricarpa* is clearly distinct from *P. capitalensis* based on sequences of the ITS region, with each species showing low levels of intraspecific variation (Hu *et al*., 2014; Okane *et al*., 2003; Rodrigues *et al*., 2004).

Significant progress in species differentiation was achieved with the introduction of multilocus DNA phylogenetic analyses. Several studies have been performed with multigene sequencing of a large number of *Phyllosticta* species (Glienke *et al*., 2011; Wang *et al*., 2012; Wulandari *et al*., 2009). Wulandari *et al*. (2009) revealed three *Phyllosticta* clades associated with citrus in Thailand using three partial DNA regions (ITS, *tef1*, *actA*), namely, *P. capitalensis*, *P. citricarpa* and *P. citriasiana.* Similarly, Wang *et al*. (2012) described *P. citrichinaensis* and distinguished two subclades within *P. citricarpa*, which were differentiated as two species in a later study (Guarnaccia *et al*., 2017a) and are described in greater detail below. In Brazil, Glienke *et al*. (2011) distinguished a new endophytic species associated with *Citrus* sp., *P*. *citribraziliensis*, combining four partial regions of DNA (ITS, *tef1*, *actA, gapdh*). The most recent study focusing on *Phyllosticta* taxonomy (Guarnaccia *et al*., 2017a) revealed two new species, namely *P*. *paracapitalensis*, from asymptomatic leaves of citrus species in Italy and Spain, and *P*. *paracitricarpa*, from leaf litter of *C*. *limon* in Greece. This study was based on the use of molecular data in which six gene regions (ITS, *actA*, *tef1*, *gapdh*, LSU, *rpb2*) were analysed.

The phylogeny of *Phyllosticta* based on the ITS and *actA* genomic loci has been reported as sufficient to differentiate most taxa, except those closely related to *P. capitalensis* (Wikee *et al*., 2013b), such as *P. paracapitalensis*, which can be distinguished by adding sequences of *tef1* and *rpb2* (Guarnaccia *et al*., 2017a). The use of a three‐gene analysis (ITS, *actA*, *tef1*) performed in a previous study by Wang *et al*. (2012) showed two poorly supported subclades within *P. citricarpa*. Highly supported independent lineages were obtained in the phylogenetic tree through the addition of a further three genomic loci (*gapdh*, LSU and *rpb2*) to confirm that the two subclades actually represent two distinct species, *P. citricarpa* and *P. paracitricarpa* (Guarnaccia *et al*., 2017a).

## Distribution and Host Associations

Since the fifth century BC, the movement of citrus plants from Asia, where citrus and CBS disease are endemic, occurred to countries around the Mediterranean Sea (Mabberley, 2004; Nicolosi, 2007; Ramon‐Laca, 2003; Wu *et al*., 2018). *Phyllosticta* spp. may therefore have been introduced to and may be present in all citrus‐growing countries.

The first report of *P. citricarpa* causing CBS disease was in Australia in the late 19th century, specifically in coastal regions of New South Wales (Benson, 1895). Subsequently, *P. citricarpa* has been recorded in many citrus‐growing areas of Australia, Africa, Asia, Central, North and South America, and Europe (Supporting Information Table S1). *Phyllosticta capitalensis* has been reported worldwide as a common endophyte of citrus and other hosts (Baayen *et al*., 2002; Wikee *et al*., 2013a). Recent studies also revealed the existence of other *Phyllosticta* species on *Citrus*. In Brazil, Glienke *et al*. (2011) described *P*. *citribraziliensis* from *Citrus* sp. In Asia, *P*. *citriasiana* (China, Thailand, Vietnam) and *P*. *citrimaxima* (Thailand) were found on *C*. *maxima* (Wikee *et al*., 2013b; Wulandari *et al*., 2009) and *P*. *citrichinaensis* was described by Wang *et al*. (2012) as a pathogen of several *Citrus* spp. in China.

In 2016, knowledge about the diversity and distribution of fungi in association with citrus was enhanced when a survey performed in citrus‐growing areas of Europe revealed the presence of different pathogens, saprobes and endophytes, including four *Phyllosticta* spp. (Crous *et al*., 2016, 2017; Guarnaccia and Crous, 2017, 2018; Guarnaccia *et al*., 2017a, 2017b; Sandoval‐Denis *et al*., 2018). Two of these species, *P*. *paracapitalensis* and *P*. *paracitricarpa*, were described as new and were isolated from fresh asymptomatic leaves in Italy and Spain, respectively, and from leaf litter of lemon in Greece. Moreover, *P*. *capitalensis* and *P*. *citricarpa* were reported by molecular analysis for the first time associated with citrus in Europe from asymptomatic leaves in Greece, Italy, Malta, Portugal and Spain, respectively, and from leaf litter in Italy, Malta and Portugal. The six‐gene phylogenetic analysis performed by Guarnaccia *et al*. (2017a) revealed that a strain previously identified as *P*. *capitalensis* from *Citrus aurantiifolia* cultivated in New Zealand (Everett and Rees‐George, 2006) also clustered with *P*. *paracapitalensis*. Similarly, two Chinese strains appearing as *P*. *citricarpa* subclade‐II *sensu* Wang *et al*. (2012) grouped with *P*. *paracitricarpa*.

Eight *Phyllosticta* species are now associated with citrus: *P. citricarpa* and *P. capitalensis* are present on all continents where citrus species are cultivated. *Phyllosticta paracapitalensis* was reported from Europe and New Zealand, while *P. paracitricarpa* is present in Asia and Europe. *Phyllosticta citrichinaensis*, *P. citriasiana* and *P. citrimaxima* were found only in Asia, and the endophyte *P. citribraziliensis* has been reported only in South America (Glienke *et al*., 2011; Guarnaccia *et al*., 2017a; Wang *et al*., 2012; Wikee *et al*., 2013b; Wulandari *et al*., 2009). Current knowledge about the distribution of these *Phyllosticta* species reveals that most of them are present in Asia, where citrus originated (Wu *et al*., 2018). However, several incursions have occurred along with the global establishment of citrus cultivation and given the long‐range dispersal of these species via infected citrus plant propagation material, it would be expected that *P*. *citricarpa* and/or other *Phyllosticta* species may be present in many citrus‐producing countries.

## Epidemiology and Symptoms

Citrus black spot disease has been reported in most of the major citrus‐producing countries, exclusively in areas with warm, summer rainfall climates (Carstens *et al*., 2012; Magarey *et al*., 2015; Martínez‐Minaya *et al*., 2018; Paul *et al*., 2005; Yonow *et al*., 2013). *Phyllosticta citricarpa* produces ascospores in pseudothecia and conidia in pycnidia as inoculum sources. In general, spore production and release occur during the rainy season (Dummel *et al*., 2015; Fourie *et al*., 2013; Kiely, 1948a; Kotzé, 1963, 1996, 2000; Reis *et al*., 2006).

Pseudothecia develop 40–180 days after leaf fall, and under favourable conditions such as alternate wetting and drying of leaves and mild to warm temperature fluctuations, inducing pseudothecium maturation and ascospore discharge (Dummel *et al*., 2015; Fourie *et al*., 2013; Hu *et al*., 2014; Huang and Chang, 1972; Kiely, 1948a; Kotzé, 1963; Lee and Huang, 1973; McOnie, 1964a; Reis *et al*., 2006; Truter, 2010).

**Figure 1:**
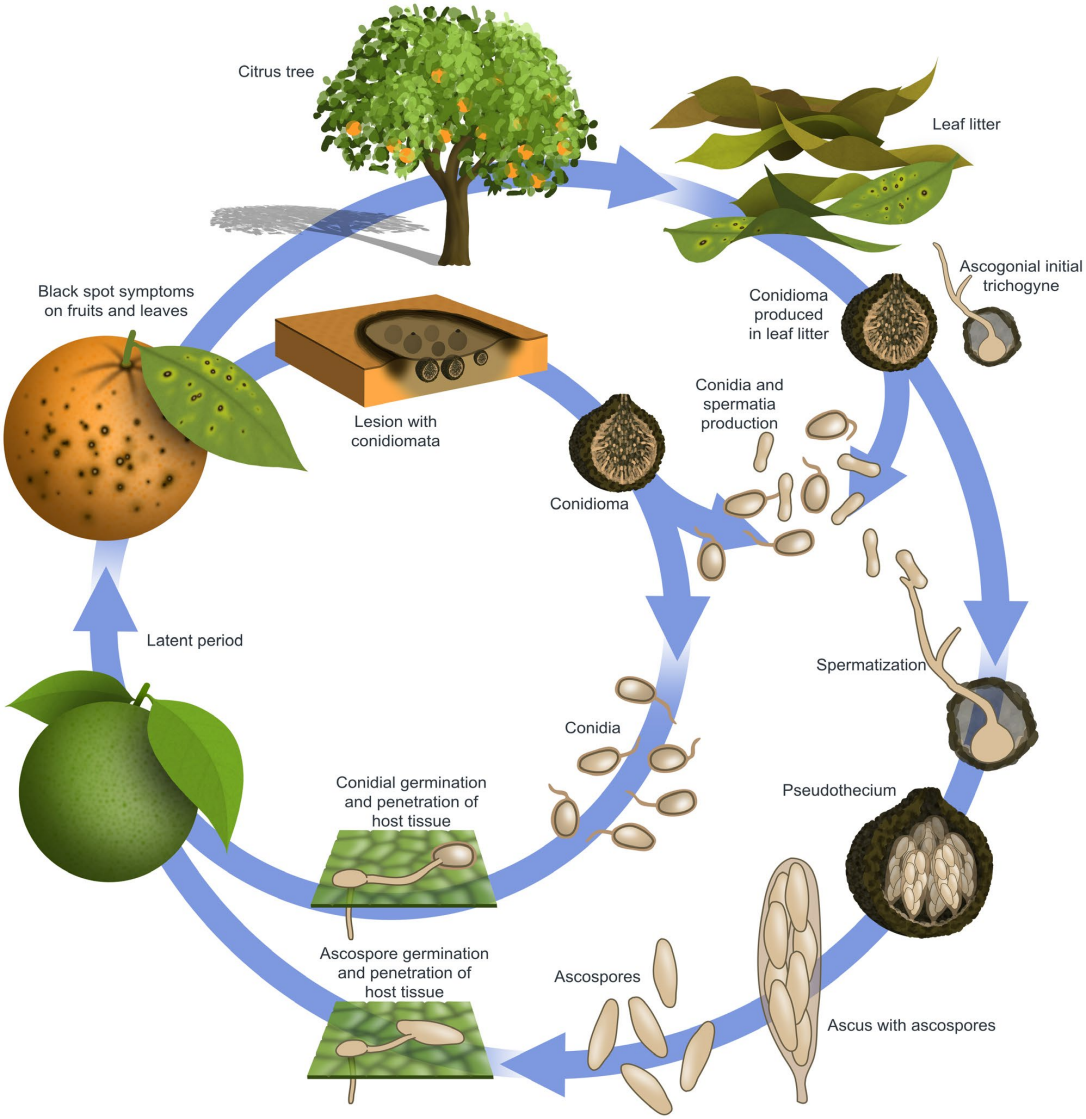
Cycle of citrus black spot disease, caused by *Phyllosticta citricarpa*.

Conidia are considered particularly important in the case of co‐existence of mature diseased fruit in a tree with young growing fruit, in areas with a large number of dead twigs and in citrus species in which hard spots with pycnidia are formed on leaves (Fig. 1) (Baldassari *et al*., 2006; Spósito *et al*., 2008, 2011). Ascospores are found only on fallen leaves (leaf litter) and not on fruit, green leaves and twigs attached to the canopy. Conidia can be found in dead twigs (not on green twigs), green leaves (mainly lemons) and diseased fruit as well as on fallen leaves (Baldassari *et al*., 2006; Kiely, 1948a; Kotzé, 1963).

An interesting case is the finding of only one mating type in the population from Florida. In this population, the primary inoculum is represented by conidia, which are related to the short‐distance dispersal of the disease (Hendricks *et al*., 2017; Wang *et al*., 2016). Thus, there is no production of pseudothecia and the asexual overwintering of the pathogen could occur endophytically as latent infections in leaves or twigs, in leaf or twig lesions, leaf litter, and on infected, out‐of‐season fruit on the tree (Kiely, 1948b; Spósito *et al*., 2007, 2011; Wager, 1952; Whiteside, 1967). Similarly, *P*. *citricarpa* strains were isolated from asymptomatic leaves and no CBS symptoms were ever found in Europe (Guarnaccia *et al*., 2017a).

Windborne ascospores are generally dispersed under field conditions over longer distances (*c*. 25 m) and are associated with the CBS spread between trees (Spósito *et al*., 2007), while conidia are dispersed over short distances (less than 80 cm) by washing‐down, being responsible for spread of the pathogen within a tree canopy (Kotzé, 1981; Spósito *et al*., 2011) or inter‐canopy (Hendricks *et al*., 2017).

Conditions required for infection by ascospores or conidia are at least 15 or 12 h leaf or fruit wetness at optimal temperatures (27 °C), respectively, while longer wetness periods are required at sub‐ or super‐optimal temperatures in the range of 15 to 35 °C (Kiely, 1948a, 1948b, 1949; Kotzé, 1963, 1981; McOnie, 1967; Noronha, 2002; Wang and Dewdney, 2014). The spores germinate and produce appressoria in the presence of moisture, and start infection by penetrating the cuticle and expanding into a small mass of mycelium between the cuticle and the epidermis wall, as a latent infection (McOnie, 1967).

Symptoms of CBS occur months later, in general after a latent period. In sweet orange fruit, false melanose lesions appear on green fruit from 40 to 110 days after inoculation, while hard spots are observed from 110 to 360 days. Some factors influence the length of the latent period, such as the inoculum concentration and fruit diameter (Frare *et al*., 2019). In addition, increasing temperatures from 20 to 27 °C when the fruit are mature can stimulate the appearance of CBS symptoms and lead to expression of a significant number of fruit lesions. High light intensity induces more fruit lesion development, thus the canopy side most exposed to light shows more symptoms. Under dry conditions CBS symptom expression may be increased. Age and stress are also linked to CBS development, since symptoms are more severe on older trees than on healthy, young trees (Kiely, 1948b; Kotze, 1981).

**Figure 2:**
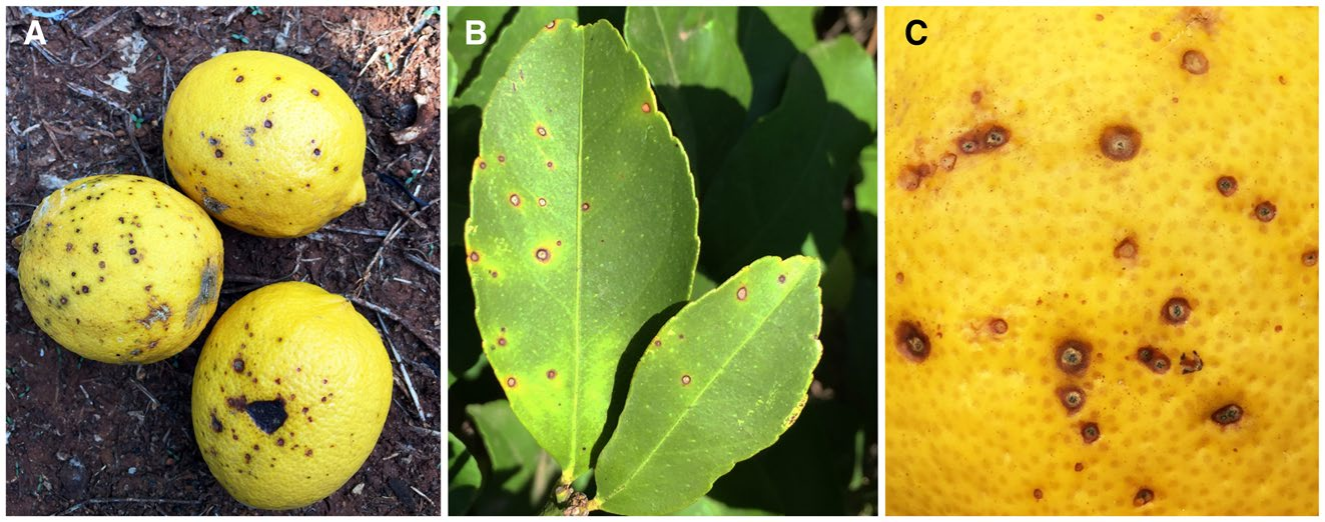
Citrus black spot symptoms caused by *Phyllosticta citricarpa*. (a) Typical hard and virulent spot with sunken necrotic lesions on *Citrus limon* fruit. (b) Hard spot lesions with a dark brown raised border on *C*. *limon* leaves. (c) Freckle spot containing pycnidia on *C*. *sinensis* rind.

*Phyllosticta citricarpa* affects fruit, leaves and twigs of several citrus hosts causing diverse symptoms (Kiely, 1948a, 1949; Kotzé, 1981, 2000; Snowdon 1990). Hard spot, characterised by sunken, pale brown necrotic lesions with a dark reddish brown raised border, often containing pycnidia, is the most common symptom (Fig. 2). Further symptom types have been described: virulent spot, which are sunken necrotic lesions without defined borders mostly on mature fruit; false melanose, consisting of small black pustules usually in a tear stain pattern; freckle, cracked or speckled spot. Leaf and twigs symptoms rarely occur on orange, mandarin and other commercial citrus species, but they are frequently present on lemons. They appear as round, small, sunken necrotic lesions with a yellow halo (Kotzé, 1981).

In Asia, *P*. *citriasiana* and *P*. *citrimaxima* were reported to cause citrus tan spot on *C*. *maxima* fruit and leaves (Wikee *et al*., 2013b; Wulandari *et al*., 2009): shallow lesions with a small central depressed grey to tan area, with a dark brown margin. This symptom usually starts appearing with fruit ripening. *Phyllosticta citrichinaensis* causes a brown spot and red‐brown protuberant freckle on citrus leaves and fruit (Wang *et al*., 2012). *Phyllosticta paracitricarpa* was isolated from fruit spots on *C*. *limon* and *C*. *sinensis* in China, and its potential pathogenicity was demonstrated only through a preliminary pathogenicity test performed on detached mature orange fruit by Guarnaccia *et al*. (2017a).

The remaining *Phyllosticta* species recovered from citrus are considered as nonpathogenic. *Phyllosticta capitalensis* has been reported from 21 different plant families (Baayen *et al*., 2002; Bezerra *et al*., 2012; Everett and Rees‐George, 2006; Glienke‐Blanco *et al*., 2002; Johnston, 1998; Meyer *et al*., 2006; Okane *et al*., 2001; Rakotoniriana *et al*., 2008; Rodrigues and Samuels, 1999; Rodrigues *et al*., 2004; Yuan *et al*., 2009) and from symptomatic and asymptomatic citrus plants (Baayen *et al*., 2002; Everett and Rees‐George, 2006; Glienke *et al*., 2011). This species was not associated with lesion formation in a pathogenicity test on detached mature sweet orange fruit (Guarnaccia *et al*., 2017a). *Phyllosticta citribraziliensis* was described from healthy citrus leaves by Glienke *et al*. (2011). *Phyllosticta paracapitalensis* is known as an endophyte from *C. limon*, *C. floridana* and *C. aurantiifolia* (Guarnaccia *et al*., 2017a).

## Sexual Reproduction and Genetics

The MAT1 mating type locus regulates sexual reproduction in ascomycetes by controlling mechanisms that lead to fertilization (Coppin *et al*., 1997; Debuchy *et al*., 2010). Two mating type idiomorphs are associated with MAT1, known as MAT1‐1, containing the *MAT1‐1‐1* gene, and MAT1‐2, containing the *MAT1‐2‐1* gene (Coppin *et al*., 1997; Debuchy and Turgeon, 2006; Debuchy *et al*., 2010; Pöggeler, 2001; Turgeon and Yoder, 2000). Numerous homothallic fungal species have been shown to possess both MAT genes, while heterothallic species have MAT1‐1 and MAT1‐2 idiomorphs in different individuals, and fertilization requires the fusion of a male (spermatium) and a female (ascogonium) elements (Alexopoulos *et al*., 1996; Coppin *et al*., 1997; Ni *et al*., 2011). Although it is not well known, the presence of spermatia and ascogonia is crucial in the *P*. *citricarpa* life cycle (Baayen *et al*., 2002; Kiely, 1948a), as well as for other members of Dothideomycetes. Kiely (1948a) was the first to reflect on the role of spermatia as male element due to the occurrence of spermatogonia prior to the formation of pseudothecial ascomata.

The heterothallic nature of *P*. *citricarpa* was recently confirmed through full sequence of target loci characterization, showing the presence of separated MAT1‐1 or MAT1‐2 idiomorphs (Amorim *et al*., 2017; Wang *et al*., 2016). Studies were performed during the last couple of decades with the aim of developing reliable methods to induce the sexual morph of *P*. *citricarpa in vitro*. The inoculation of a single mycelium plug or of a mycelial and spore suspension on a medium containing lemon leaf and malt extracts was reported as successful by Moran Lemir *et al*. (2000). However, this process was not reproducible during subsequent studies (Baldassari *et al*., 2008; Wang *et al*., 2016). Amorim *et al*. (2017) conducted *in vitro* crossings with isolates of different mating types on various media and incubated plates at different conditions, but did not observe any sexual structures. The first successful and reproducible mating of *P. citricarpa* in culture was reported by Tran *et al*. (2017), involving direct physical contact between two isolates of complementary mating types. The spermatia function as male gametes and their fertilization of receptive organs during sexual reproduction of *P. citricarpa* via spermatization was demonstrated. Spermatia were shown to be incapable of germinating and forming colonies on media, demonstrating their role in sexual reproduction rather than vegetative growth. This study confirmed that the mating type genes of *P. citricarpa* are functional and that sexual reproduction can occur under laboratory conditions. Furthermore, Tran *et al*. (2017) conducted multilocus genotyping using simple‐sequence repeat (SSR) markers to determine the genotypes and hybrid nature of the cultures derived from ascospores obtained *in vitro*. These results demonstrate that new recombinant genotypes were formed in the F_1_ progeny, confirming that genetic recombination occurs. Tran *et al*. (2018) also tested the pathogenicity of *P*. *citricarpa* ascospores, obtaining typical CBS symptoms on Troyer citrange leaves and Murcott tangor fruit with ascospores produced *in vitro* from characterized *P*. *citricarpa* isolates, confirmed with recovering and genotyping the inoculated recombinant ascospore isolates from obtained symptoms.  These recent findings are crucial for new research projects aimed at understanding pathogenicity, as well as management of diseases caused by *Phyllosticta* species.

In Australia, Brazil, China and South Africa, MAT genotyping of populations revealed an almost equal mating type distribution, confirming the occurrence of sexual reproduction in these populations (Amorim *et al*., 2017; Carstens *et al*., 2017; Tran *et al*., 2017; Wang *et al*., 2016; Zhang *et al*., 2015). Interestingly, in Florida in the USA, only one mating type has been identified (Carstens *et al*., 2017; Wang *et al*., 2016). Both MAT1‐1 and MAT1‐2 containing isolates occur in Europe, but both mating types were not recovered together in the same geographic location, nor were pseudothecial ascomata ever observed (Guarnaccia *et al*., 2017a).

The first SSR markers for *P. citricarpa* were developed by Wang *et al*. (2016) from two published genome sequences of *P. citricarpa* and consist of seven polymorphic loci. One population from Australia and one from Florida were genotyped using these markers. Two to four alleles per locus were identified in the Australian population, and 11 multilocus genotypes (MLGs) among 24 Australian isolates. The populations from Florida consisted of a single MLG, confirming the results obtained in previous studies based on MAT genotyping (Wang *et al*., 2016; Zhang *et al*., 2015). Population genetic inferences were strengthened by the development of eight new polymorphic SSR markers by Carstens *et al*. (2017), which were added to the seven markers by Wang *et al*. (2016). Thus, a total of 15 SSR markers was used to genotype several populations from Australia, Brazil, China, Florida (USA) and South Africa (Carstens *et al*., 2017). The high level of gene and genotypic diversity within *P. citricarpa* populations of China and Australia, as well as the presence of several private alleles, demonstrated a longer evolutionary history with the citrus host in these countries, compared to South Africa, Brazil and the USA. The US population was confirmed to be clonal (Carstens *et al*., 2017). Populations from Australia, Brazil, South Africa and the USA shared an MLG, suggesting a long‐distance human‐mediated dispersal. Moreover, the clonal mating type (*MAT1‐2‐1*) present in the US population suggested a human introduction, with subsequent asexual reproduction of the pathogen in Florida (Carstens *et al*., 2017; Hendricks *et al*., 2017; Wang *et al*., 2016).

Another recent study based on the same 15 SSR markers used by Carstens *et al*. (2017) revealed that the *P. citricarpa* populations from Italy and Malta versus Portugal represented two separate clones, differing from each other in their MLGs and mating type (Guarnaccia *et al*., 2017a). The origin of *P. citricarpa* in Europe is still unclear because these populations also differed from one another in their connectivity and differentiation level from the other populations from Australia, Brazil, China, South Africa and the USA. The genotyping performed in that study showed that the populations from Portugal and Australia were more strongly connected to each other than to other populations, whilst very little connectivity was evident between the Portuguese population and those from the other continents, including the population from Italy and Malta. Similarly, the Italy/Malta population appeared to be distinct from the other known populations. These results suggest at least two separate introductions into Europe.

## Genomics

**Figure 3:**
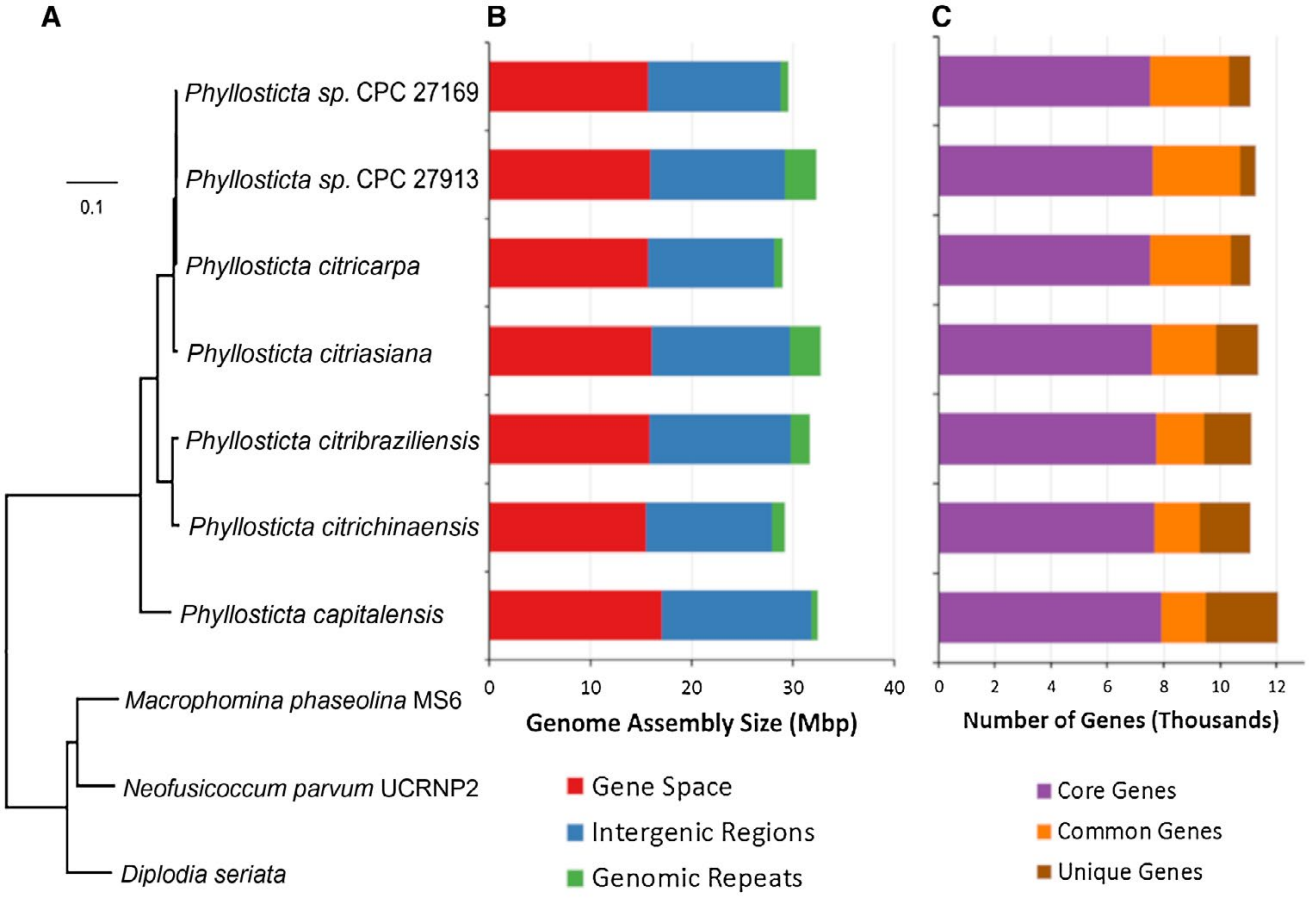
Phylogeny, assembly characteristics and gene composition of *Phyllosticta* genomes. (A) Genome based mid‐point rooted phylogenetic tree using 3455 single copy proteins identified using Markov Cluster Algorithm (mcl 10‐148, 1.008). Protein sequences were aligned with MAFFT (v. 7.123b) and the resulting alignment was cleaned using Gblocks 0.91b. The final alignment had 177824 distinct alignment patterns and was used for tree building using RAxML (v. 7.6.3) with 100 rapid bootstrap inferences, GAMMA model of rate heterogeneity and ML estimate of *α*‐parameter. All nodes showed 100% bootstrap support. (B) Genome size and repeat content. Predicted genes (CDS) covers about half of the assembled genome while genomic repeats cover between 2% and 10% of these assemblies. (C) Number of predicted genes. Core Genes are found in all *Phyllosticta* genomes, Common Genes are found in more than one *Phyllosticta* genome while Unique Genes are found only in one genome.

Seven genomes and transcriptomes of *Phyllosticta* spp., including two strains of *P. citricarpa*, have been sequenced as part of the 1000 Fungal Genomes initiative of the United States Department of Energy Joint Genomics Institute (JGI; http://1000.fungalgenomes.org) (Table 1). The QIAGEN Genomic‐tip 100/G (QIAGEN Benelux B.V., Venlo, Netherlands) and the QIAGEN RNeasy Midi kit have been used, respectively, for DNA and RNA isolation. All of these genomes, except for *P. citriasiana* (strain CBS 120486) were sequenced using PacBio technology and assembled with Falcon v. 1.8.8 (https://github.com/PacificBiosciences/FALCON). The genome of *P. citriasiana* was sequenced using the Illumina platform and assembled with AllPathsLG release v. R49403 (Gnerre *et al*., 2011). All transcriptomes were sequenced using Illumina and assembled with Trinity v. 2.3.2 (Grabherr *et al*., 2011). All genomes were annotated using the JGI Annotation Pipeline (Grigoriev *et al*., 2014). The average *Phyllosticta* genome is about 30.9 Mbp with about 5.2% repeats and *c*.11 300 genes (Table 1, Fig. 3). Core Eukaryotic Genes Mapping Approach (CEGMA) (Parra *et al*., 2007) analysis showed that these assemblies capture more than 99.5% of conserved eukaryotic genes. In all these genomes, >70% of genes had more than one exon (median = 2), similar to other Dothideomycetes, with an average gene density of 365.33 Mbp. Of the 11 300 genes in a *Phyllosticta* genome, more than two‐thirds (67.6%) are shared with all other *Phyllosticta* strains in this study and an additional 20% are found in more than one *Phyllosticta* genome. The largest families include transporters, protein kinases and transcription factors. On average, 12% of these genes were unique to each species in our study (Fig. 3), with the largest fraction of species‐specific genes in *P. capitalensis* at the base of the *Phyllosticta* tree, and fewer differences within the group of two *P*. *citricarpa* isolates and *P*. *paracitricarpa*. However, further studies are needed to explore the variation within the *P*. *citricarpa* and *P*. *paracitricarpa* populations.

**Table 1:** Assembly statistics, gene content and annotation characteristics of seven *Phyllosticta* spp. genomes sequenced for this study.

	*P. capitalensis* CBS 128856	*P. citriasiana* CBS 120486	*P. citribraziliensis* CBS 100098	*P. citrichinaensis* CBS 130529	*P. paracitricarpa* CBS 141357	*P. citricarpa* CBS 127454	*P. citricarpa* CBS 141350
Assembly length	32 461 131	32 696 106	31 670 975	29 162 704	29 529 839	28 952 665	32 267 666
# contigs (scaffolds)	14	741 (133)	32	25	134	152	82
Contig (scaffold) N50	5	56 (14)	7	4	23	23	13
Contig (scaffold) L50, bp	2 860 346	163 917 (807 147)	1 720 616	2 710 567	510 184	440 231	900 945
% GC, ignoring Ns	54.58	53.05	54.17	55.07	54.35	54.6	52.56
% repeats	2.07	9.24	6.16	4.34	2.62	2.76	9.46
Gene density (Mbp)	371.61	347.69	350.51	380.35	375.25	382.97	348.93
# genes	12 063	11 368	11 101	11 092	11 081	11 088	11 259
% spliced	70.73	72.69	74.17	71.41	72.12	72.75	71.92
% CEGMA coverage	99.78	99.78	99.78	100	99.78	99.56	100
# unique domains hit (HMMpfam)	3764	3742	3741	3713	3692	3700	3733

**Figure 4:**
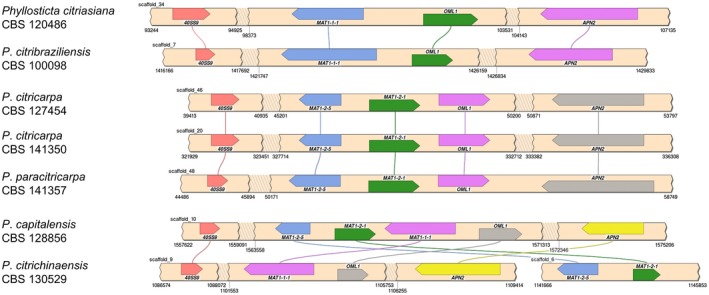
Syntenic relationships between mating type loci in different species of *Phyllosticta*. For each genome, the regions of the scaffolds containing mating type genes are illustrated as beige strips. Shaded breaks in the strips indicate larger genomic regions that do not contain our mating type genes, which are not visualized for clarity and brevity. The scaffold numbers and the relevant positions are annotated. Coloured boxes on the scaffolds indicate mating type genes, and the pointed end refers to the 3' end of the gene. Boxes with the same colour indicate the same mating type gene. Lines link the boxes to indicate their positions in the different genomes. Together, this figure visualizes the rearrangement of mating type loci across the *Phyllosticta* genomes.

In order to provide a foundation for future studies, the mating type loci of the seven *Phyllosticta* genomes have been analysed with the aim of identifying which mating type(s) are present in *Phyllosticta* species and to characterize the mating type genes. Based on a comparison of the genes known to be in *P. citricarpa* (Amorim *et al*., 2017; Wang *et al*., 2016), the 40SS9, PH Domain, MAT1‐1‐1, MAT1‐2‐9, MAT1‐2‐1, OML1 and APN2 protein sequences (see Table S3 for accessions) have been selected. Then, based on translated nucleotide blast (Altschul *et al*., 1997), the scaffolds were identified in each assembly that contain fragments of the mating type locus genes. Both strains of *P. citricarpa* (European and Australian strains) and the *P. paracitricarpa* strain have heterothallic idiomorphs containing the *MAT1‐2‐1* gene. In contrast, *P. citriasiana* and *P. citribraziliensis* strains have heterothallic idiomorphs containing the *MAT1‐1‐1* gene. *Phyllosticta citrichinaensis* is homothallic, containing the full MAT1‐1 and MAT1‐2 loci on different scaffolds. Finally, *P*. *capitalensis* seems to contain a hybrid of both MAT1‐1 and MAT1‐2 loci, with the *MAT1‐2‐1* and *MAT1‐1‐1* co‐linearly present between the *40SS9* and *OML1* genes. The scaffolds with the MAT locus fragments were visualized with SimpleSynteny (Veltri *et al*., 2016) (Fig. 4).

In addition, new *Phyllosticta*‐specific mating type primers (Table 2) have been designed and a PCR method developed for rapid identification of the *MAT1‐1‐1* and *MAT1‐2‐1* genes in *P. capitalensis*, *P. citriasiana*, *P. citribraziliensis*, *P. citricarpa*, *P. citrichinaensis* and *P. paracitricarpa* strains (Supporting Information Fig. S1). The primer pair MAT111deg‐F2 and MAT111deg‐R3 amplified a fragment of approximately 1010 bp, whilst the primer pair MAT121deg‐F1 and MAT121deg‐R1 amplified a 300‐bp fragment of several tested isolates (Supporting Information Table S2).

**Table 2:** Primers designed and used in this study.

Primer	Sequence (5'–3')	Description
MAT111degF2	GCTCTCAACTCTTTCATGGC	*Phyllosticta* spp. *MAT1‐1‐1* specific
MAT111degR3	TGGYKCGYYGCATCACGC	*Phyllosticta* spp. *MAT1‐1‐1* specific
MAT121degF1	AACAYRTCRARGCYCCGG	*Phyllosticta* spp. *MAT1‐2‐1* specific
MAT121degR1	YAABCCTGGRTTYTCCATCG	*Phyllosticta* spp. *MAT1‐2‐1* specific

## Disease Management

Citrus tan spot caused by *P*. *citriasiana* and *P*. *citrimaxima* was only recently described and their economic importance is uncertain. Likewise, the economic importance of *P. paracitricarpa* is unknown. Control of these *Phyllosticta* species has not been studied, but it is likely to be effectively controlled by CBS management measures.

The management of CBS starts with planting disease‐free citrus trees, since infected propagation material is the most effective means of spreading *P. citricarpa* to new areas (Doidge, 1929; Kiely, 1948b; Kotze, 1981; Marchionatto, 1926; McOnie, 1964b; Silva‐Junior *et al*., 2016a; Wager, 1952). Regulatory measures are imposed by some countries to prevent the import and spread of *P. citricarpa* on infected propagation material into CBS‐free areas (EFSA, 2014; Kotzé, 1981). In addition, the movement of leaf litter from infected orchards through vehicle/machine movement is also important (Dewdney *et al*., 2018; Silva‐Junior *et al*., 2016a). Citrus fruit is not considered to be a realistic pathway for spread of *P. citricarpa* to new areas (USDA APHIS, 2010) for the following reasons: (i) the airborne ascospores cannot be produced on fruit, (ii) pycnidia are only produced in certain fruit lesion types (Brentu *et al*., 2012; FAO, 2014; Kotzé, 2000; Marques *et al*., 2012; OEPP/EPPO, 2009; Wager, 1952) and conidia are short‐lived with low germination ability (Kiely, 1948b), (iii) conidium dispersal from fruit lesions is by means of short‐distance (<1 m) wash‐down dispersal (Kiely, 1948b; McOnie, 1965; Spósito *et al*., 2008, 2011; Whiteside, 1967), (iv) standard packhouse treatments and cold storage effectively control *P. citricarpa* infections (Korf *et al*., 2001; Lucon *et al*., 2010; Rappussi *et al*., 2009, 2011; Schreuder *et al*., 2018; Seberry *et al*., 1967; Yan *et al*., 2016), and CBS lesions on fruit or discarded peel segments have a very low reproductive potential (Korf *et al*., 2001; Schreuder *et al*., 2018; Schutte *et al*., 2014), and (v) fallen leaves are not susceptible to infection (Truter *et al*., 2007). Inter‐state movement of commercial packhouse‐treated fruit from CBS present to CBS‐absent areas is therefore permitted in the USA, in line with their Pest Risk Analysis conclusion that fruit is not a realistic pathway (USDA APHIS, 2011). In European Union countries, where CBS disease has not been recorded, regulatory measures have been imposed on the import of citrus fruit from countries where CBS is present (EFSA, 2014). However, the technical justification for these measures has been contested (CBS Expert Panel, 2013, 2014, 2015).

Different chemical and cultural control measures are used for CBS management (Dewdney *et al*., 2018; Kotzé, 2000; Silva‐Junior *et al*., 2016a). The most effective strategy for control of CBS is the fungicide application during the period of fruit susceptibility (Lanza *et al*., 2018; Makowski *et al*., 2014; Schutte *et al*., 2003). The main fungicides used are strobilurins (quinone outside inhibitors, QoI), dithiocarbamates and fixed copper (multisite activity), and methyl benzimidazole carbamate (MBC), which may be applied singly or in mixtures with mineral oil (Dewdney *et al*., 2018; Kellerman and Kotze, 1977; Kotze, 1981, 2000; Miles *et al*., 2004; Schutte *et al*., 2003, 2012; Silva‐Junior *et al*., 2016a, 2016b). Sprays of QoI fungicides may reduce CBS symptoms by almost 100%, becoming one of the most valuable solutions for disease control (Dewdney *et al*., 2018; Fogliata *et al*., 2011; Miles *et al*., 2004; Schutte *et al*., 2003; Silva‐Junior *et al*., 2016a). The mode of action of QoI is highly specific; however, the presence of an intron in the cytochrome b gene reduces the risk of the G143A mutation, which causes QoI resistance (Hincapie *et al*., 2014; Stammler *et al*., 2013). Nonetheless, the number of QoI sprays per season should be restricted to prevent resistance development by another mechanism (Dewdney *et al*., 2018; Silva Junior *et al*., 2016a, 2016b).

In countries such as South Africa and Australia, the applications for control of CBS are restricted to the critical period of *P. citricarpa* infection, from petal fall stage, in October, to 120–150 days later, January–February (Kotzé 1981; Kiely, 1948b; Miles *et al*., 2004; Schutte *et al*., 1997, 2003). Typically, two to five spray rounds (dependent on citrus type and climate suitability of the production region for CBS) of mancozeb or copper‐based fungicides alone, or in mixture with MBC or QoI plus oil, are applied in South Africa, using high volumes from 6000 to 16 000 L/ha (Moyo *et al*., 2018; Schutte *et al*., 1997, 2003, 2012). In Brazil, where conditions are much more favourable for CBS (Magarey *et al*., 2015), a longer period of fruit protection is required (Lanza *et al*., 2018; Silva‐Junior *et al*., 2016a, 2016b). Two copper sprays are performed after petal fall, between September and November with a 21‐ to 28‐day interval, not only for CBS, but also to control other citrus diseases, followed by two to four QoI applications, performed with a 35‐ to 42‐day interval. This programme protects the fruit for 180–220 days until March or up to May. The spray volume is around 75 mL of spray mixture/m^3^ of the tree canopy, based on the tree‐row‐volume (TRV) concept (Lanza *et al*., 2018; Silva‐Junior *et al*., 2016a, 2016b). In the USA, the control programme has been based on fungicide efficacy results from other countries. Applications of copper and QoI fungicides are recommended from early May to mid‐September, but if favourable conditions occur in April, earlier sprays are recommended (Dewdney *et al*., 2018; Hincapie *et al*., 2014). Miles *et al*. (2004) demonstrated CBS control using the host defence promoter acibenzolar‐S methyl in the field, but it was not as effective as the registered fungicides.

In addition to chemical control, cultural control measures may be used to reduce the amount of *P. citricarpa* inoculum (Bellotte *et al*., 2009, 2013; Schutte and Kotzé, 1997), such as the removal of leaf litter with machines (Bellotte *et al*., 2009; Scaloppi *et al*., 2012; Spósito *et al*., 2011; Truter, 2010), the acceleration of leaf litter decomposition with urea, ammonium sulphate, sugarcane bagasse (Bellotte *et al*., 2009; Dewdney *et al*., 2018; Kotzé, 1981; van Bruggen *et al*., 2017), the mulching with plants that grow between rows of orchards to cover leaf litter (Bellotte *et al*., 2013; Schutte and Kotzé, 1997), the pruning of dead twigs (Silva *et al*., 2017; Silva‐Junior *et al*., 2016a), irrigation and balanced nutrition (Calavan, 1960; Dewdney *et al*., 2018; Kotzé, 1981), and the harvesting for optimal fruit quality and prevention of overlapping fruit sets (Kotzé, 1981; Spósito *et al*., 2008, 2011). Biological control of CBS with fungi, including *P. capitalensis*, and bacteria have shown inhibitory effect against *P. citricarpa in vitro* and *in vivo* (Almeida, 2009; Kupper *et al*., 2011; Pena *et al*., 2017; Santos *et al*., 2016; Tran *et al*., 2019); however, there is no biocontrol agent that controls CBS with fungicide‐like efficiency under field conditions. Genetic methods may improve the CBS resistance of citrus. Rodriguez *et al*. (2018) produced transgenic sweet orange trees with reduced production of d‐limonene in the fruit flavedo and enhanced resistance against *P. citricarpa*, but these materials need to be assessed under field conditions.

Postharvest treatments aimed at preventing decay and maintaining fruit quality have a strong effect on the CBS pathogen. Low temperatures reduce the postharvest development of symptoms on fruit (Agostini *et al*., 2006; Brodrick and Rabie, 1970) and standard packhouse treatments reduce the viability of the fungus in fruit lesions by three‐ to seven‐fold (Korf *et al*., 2001). Schreuder *et al*. (2018) demonstrated that the combination of standard packhouse fungicide treatments aimed at green mould or sour rot control (including pre‐packhouse drench, packhouse dip and brush application of a wax coating) followed by cold storage (common shipping protocol for exported fresh fruit) consistently showed moderate to high levels of control of CBS in lemons and oranges. Several other studies also reported control of latent *P. citricarpa* infections through application of various chemical or biological postharvest treatments (Lucon *et al*., 2010; Rappussi *et al*., 2009, 2011; Seberry *et al*., 1967; Yan *et al*., 2016). On the contrary, Agostini *et al*. (2006) reported no significant control with the postharvest fungicides evaluated in their study.

## Conclusions and Future Research

Considerable taxonomic confusion has characterized studies about *Phyllosticta* spp. in the past, especially regarding the common mistake to distinguish *P. capitalensis* and *P. citricarpa* associated with symptoms on fruit, or the identification of *P. citriasiana* and *P. citrichinaensis.* After the advent of DNA‐based characterization, the taxonomy and classification of these species were clarified, offering new data and methods to improve rapid detection. Many genomes of several important plant pathogenic and endophytic fungi such as various *Phyllosticta* species have been sequenced. The genomes of *P. citricarpa* and *P. capitalensis* were already sequenced by Wang *et al*. (2016). The citrus pathogens *P. citriasiana*, *P. citrichinaensis* and *P. paracitricarpa*, and also the endophytic species *P. citribraziliensis*, are also now available for study.

Although the sexual morph of *P*. *citricarpa* was known (Wang *et al*., 2016), it was only recently successfully induced *in vitro* by Tran *et al*. (2017) through outcrossing of isolates carrying complementary MAT idiomorphs, thus demonstrating the heterothallic behaviour of *P*. *citricarpa*. In the same study, the sexual function of spermatia as male gametes in the life cycle of *P*. *citricarpa*, and the fertilization in this fungus occurring via spermatization, were also demonstrated (Tran *et al*., 2017). However, no research has to date focused on the sexual morph of the other *Phyllosticta* spp. associated with citrus, except for *P*. *capitalensis* that was demonstrated as being homothallic by Wang *et al*. (2016). Based on the whole genome analysis of several strains in this study, we identified *P*. *citriasiana*, *P*. *citribraziliensis* and *P*. *paracitricarpa* as heterothallic, and *P*. *citrichinaensis* as homothallic. We partially sequenced the *MAT1‐1‐1* and *MAT1‐2‐1* genes present in additional isolates of all the tested species by using new sets of specific primers designed in this study.

Future studies on the global distribution of mating types of *P*. *citricarpa* at the lesion, fruit and tree level, and movement of spermatia on the plant at the infection stage, are necessary to study their primary role in ascospore production, as well as to explain the occurrence of sexual reproduction of the fungus on leaf litter under orchard conditions. Such data would clarify the role of all the spore types in the disease cycle of CBS, aiding the development of a good and fast method for pathogenicity testing in order to screen various chemicals and/or biological agents. Larger populations of *P*. *citriasiana*, *P. citrichinaensis* and *P. paracitricarpa* are required to make it possible to obtain more details about the distribution of mating types for other pathogenic *Phyllosticta* spp.

The available genomic sequences for six species of the genus *Phyllosticta* provide an important resource to gain a better understanding regarding the distribution, sexual morph, rapid detection and marker development of these important species. However, more *P*. *citricarpa* genomes of both MAT idiomorphs and from diverse areas are essential. Also, the sequencing of *P*. *paracapitalensis*, which was unknown when the strains used in this study for the genome sequencing were selected, is still absent.

Further studies must be conducted on a wider global selection of *P. citricarpa* strains to improve our knowledge of its host association and distribution. A broader sampling is required to collect additional populations from Europe, Asia and Oceania for more detailed population studies. Population genetic inferences were strengthened by the development of several polymorphic SSR markers (Carstens *et al*., 2017; Wang *et al*., 2016), but a complete picture of the introduction and global movement pathways has not yet emerged. Therefore, more informative markers are needed to examine the population structure and migration pathways of *P*. *citricarpa* and other *Phyllosticta* spp. The development of new informative markers is crucial to accurately characterize clones and to track the global movement and introductions of *P*. *citricarpa* along with plant material from Asia, where *Citrus* is endemic, to other continents. These studies can also provide information on pathogenicity mechanisms, which remain very poorly understood.

## Supporting information


**Fig. S1** Different *Phyllosticta* isolates screened using the MAT111deg‐F2 and MAT111deg‐R3 primers (1010‐bp fragment; top part of both gel photos), and the same *Phyllosticta* isolates screened with the MAT121deg‐F1 and MAT121deg‐R1 primers (300‐bp‐fragment; lower part of both gel photos).

**Table S1** Geographical distribution of *Phyllosticta* *citricarpa.*

**Table S2** Collection and sequencing details of isolates included in this study.

**Table S3** Accession numbers of mating type loci genes used in the synteny visualization.
