# Supplementary material for: Phyllosticta citricarpa and sister species of global importance to Citrus
Source: Mol Plant Pathol. 2019 Sep 11;20(12):1619–35. doi: 10.1111/mpp.12861 (PMC6859488; doi:10.1111/mpp.12861)

**Fig. S1**. Different *Phyllosticta* isolates screened using the MAT111deg-F2 and MAT111deg-R3 primers (1,010-bp-fragment; top part of both gel-photo), and the same *Phyllosticta* isolates screened with the MAT121deg-F1 and MAT121deg-R1 primers (300-bp-fragment; lower part of both gel-photo).


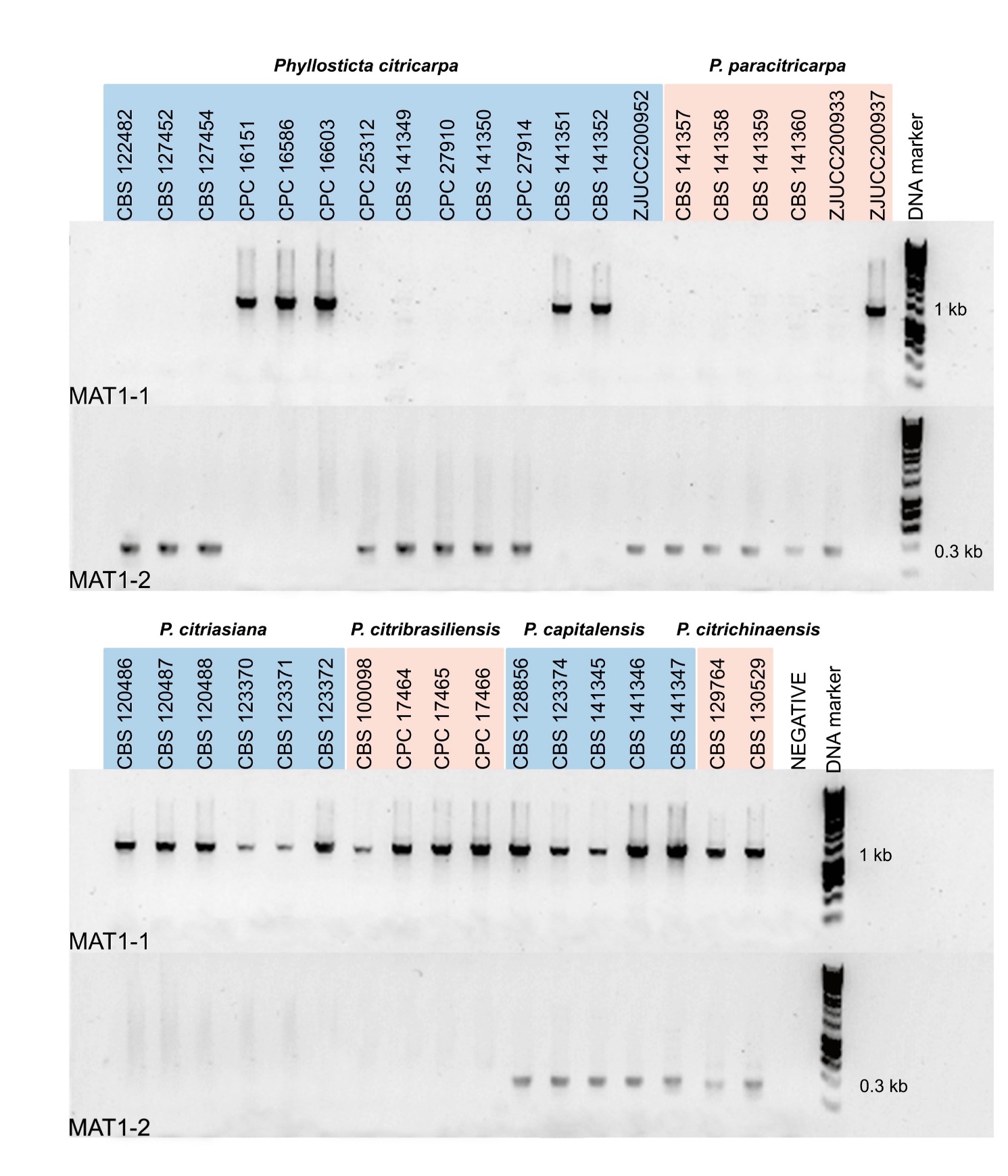

Supplement: Supplementary file 1 — Fig. S1 Different Phyllosticta isolates screened using the MAT111deg‐F2 and MAT111deg‐R3 primers (1010‐bp fragment; top part of both gel photos), and the same Phyllosticta isolates screened with the MAT121deg‐F1 and MAT121deg‐R1 primers (300‐bp‐fragment; lower part of both gel photos). [file MPP-20-1619-s001.docx]
