# Supplementary material for: Phyllosticta citricarpa and sister species of global importance to Citrus
Source: Mol Plant Pathol. 2019 Sep 11;20(12):1619–35. doi: 10.1111/mpp.12861 (PMC6859488; doi:10.1111/mpp.12861)
Supplement: Supplementary file 2 — Table S1 Geographical distribution of Phyllosticta citricarpa. [file MPP-20-1619-s002.docx]

**Table S1.** Geographical distribution of *Phyllosticta* *citricarpa*.

| **Continent** | **Country/Region** | **References** |
| --- | --- | --- |
| Africa | Angola, Ghana, Kenya, Mozambique, Namibia, Nigeria, South Africa, Swaziland, Uganda, Zambia, Zimbabwe | Doidge, 1929; Kotzé, 1981, 2000; Baayen *et al*., 2002; Reeder *et al*., 2009; Brentu *et al*., 2012; Bassimba *et al*., 2018. |
| Asia | Bhutan, China, India, Indonesia, Philippines, Taiwan | Brodrick, 1969; Zheng, 1983; Kotzé, 2000; Das *et al*., 2018. |
| Oceania | Australia | Benson, 1895; Cobb, 1897; Kiely, 1948a,b. |
| Central America | Cuba | CABI/EPPO, 2012; Calavan, 1960. |
| South America | Argentina, Brazil, Uruguay | Robbs *et al*., 1980; Foguet *et al*., 1985; Kotzé, 2000; European Union, 2000; Paul *et al*., 2005. |
| North America | United States | Schubert *et al.*, 2012; Zavala *et al.*, 2014. |
| Europe | Italy, Malta, Portugal | Guarnaccia *et al*., 2017a. |
