# Supplementary material for: Phyllosticta citricarpa and sister species of global importance to Citrus
Source: Mol Plant Pathol. 2019 Sep 11;20(12):1619–35. doi: 10.1111/mpp.12861 (PMC6859488; doi:10.1111/mpp.12861)
Supplement: Supplementary file 3 — Table S2 Collection and sequencing details of isolates included in this study. [file MPP-20-1619-s003.docx]

**Table S2**. Collection and sequencing details of isolates included in this study.

* CPC: Culture collection of P.W. Crous, housed at Westerdijk Fungal Biodiversity Institute; CBS: Westerdijk Fungal Biodiversity Institute, Utrecht, the Netherlands; ZJUCC: Zhejiang University Culture Collection, China.

| **Species** | **Strain number*** | **Host** | **Country** |
| --- | --- | --- | --- |
|  |  |  |  |
| *Phyllosticta capitalensis* | CBS 123374 | *Citrus aurantium* | Thailand |
|  | CBS 128856 | *Stanhopea graveolens* | Brazil |
|  | CBS 141345 | *Citrus aurantifolia* | Italy |
|  | CBS 141346 | *Citrus medica* | Italy |
|  | CBS 141347 | *Citrus limon* | Malta |
| *P. citriasiana* | CBS 120486 | *Citrus maxima* | Thailand |
|  | CBS 120487 | *Citrus maxima* | China |
|  | CBS 120488 | *Citrus maxima* | Thailand |
|  | CBS 123370 | *Citrus maxima* | Vietnam |
|  | CBS 123371 | *Citrus maxima* | Vietnam |
|  | CBS 123372 | *Citrus maxima* | Vietnam |
| *P. citribraziliensis* | CBS 100098 | *Citrus limon* | Brazil |
|  | CPC 17464 | *Citrus* sp. | Brazil |
|  | CPC 17465 | *Citrus* sp. | Brazil |
|  | CPC 17466 | *Citrus* sp. | Brazil |
| *P. citricarpa* | CBS 122482 | *Citrus sinensis* | Zimbabwe |
|  | CBS 127452 | *Citrus reticulata* | Australia |
|  | CBS 127454 | *Citrus limon* | Australia |
|  | CBS 141349 | *Citrus limon* | Italy |
|  | CBS 141350 | *Citrus sinensis* | Malta |
|  | CBS 141351 | *Citrus sinensis* | Portugal |
|  | CBS 141352 | *Citrus sinensis* | Portugal |
|  | CPC 16151 | *Citrus* sp. | South Africa |
|  | CPC 16586 | *Citrus limon* | Agentina |
|  | CPC 16603 | *Citrus limon* | Uruguay |
|  | CPC 25312 | *Citrus sinensis* | Florida, US |
|  | CPC 27910 | *Citrus limon* | Italy |
|  | CPC 27914 | *Citrus sinensis* | Malta |
|  | ZJUCC200952 | *Citrus reticulata* | China |
| *P. citrichinaensis* | CBS 129764 | *Citrus reticulata* | China |
|  | CBS 130529 | *Citrus maxima* | China |
| *P. paracitricarpa* | CBS 141357 | *Citrus limon* | Greece |
|  | CBS 141358 | *Citrus limon* | Greece |
|  | CBS 141359 | *Citrus limon* | Greece |
|  | CBS 141360 | *Citrus limon* | Greece |
|  | ZJUCC200933 | *Citrus sinensis* | China |
|  | ZJUCC200937 | *Citrus sinensis* | China |
