# Supplementary material for: Phyllosticta citricarpa and sister species of global importance to Citrus
Source: Mol Plant Pathol. 2019 Sep 11;20(12):1619–35. doi: 10.1111/mpp.12861 (PMC6859488; doi:10.1111/mpp.12861)
Supplement: Supplementary file 4 — Table S3 Accession numbers of mating type loci genes used in the synteny visualization. [file MPP-20-1619-s004.docx]

**Table S3**. Accession numbers of mating type loci genes used in the synteny visualization.

| **Name** | **Accession used for Synteny visualization** |
| --- | --- |
| APN2 | AQX35417.1 |
| OML1 | AMP46562.1 |
| 40SS9 | AMJ39451.1 |
| MAT-1-1-1 | AQX35418.1 |
| MAT-1-2-1 | AMP46551.1 |
| MAT-1-2-9 | AQX35421.1 |
| PH–Domain | AMJ39457.1 |
